# Inhibition of Microprocessor Function during the Activation of the Type I Interferon Response

**DOI:** 10.1016/j.celrep.2018.05.049

**Published:** 2018-06-13

**Authors:** Jeroen Witteveldt, Alasdair Ivens, Sara Macias

**Affiliations:** 1 Institute of Immunology and Infection Research, School of Biological Sciences, University of Edinburgh, Edinburgh, EH9 3FL, UK

**Keywords:** microRNAs, microprocessor, DGCR8, Drosha, type I interferon, dsRNA, antiviral

## Abstract

Type I interferons (IFNs) are central components of the antiviral response. Most cell types respond to viral infections by secreting IFNs, but the mechanisms that regulate correct expression of these cytokines are not completely understood. Here, we show that activation of the type I IFN response regulates the expression of miRNAs in a post-transcriptional manner. Activation of IFN expression alters the binding of the Microprocessor complex to pri-miRNAs, reducing its processing rate and thus leading to decreased levels of a subset of mature miRNAs in an IRF3-dependent manner. The rescue of Microprocessor function during the antiviral response downregulates the levels of IFN-β and IFN-stimulated genes. All these findings support a model by which the inhibition of Microprocessor activity is an essential step to induce a robust type I IFN response in mammalian cells.

## Highlights

• Antiviral response transiently impairs Microprocessor function
• Microprocessor binding and processing efficiency is regulated by IFN response
• Regulation of Microprocessor activity is essential for robust IFN expression

## Graphical Abstract

**Figure:**
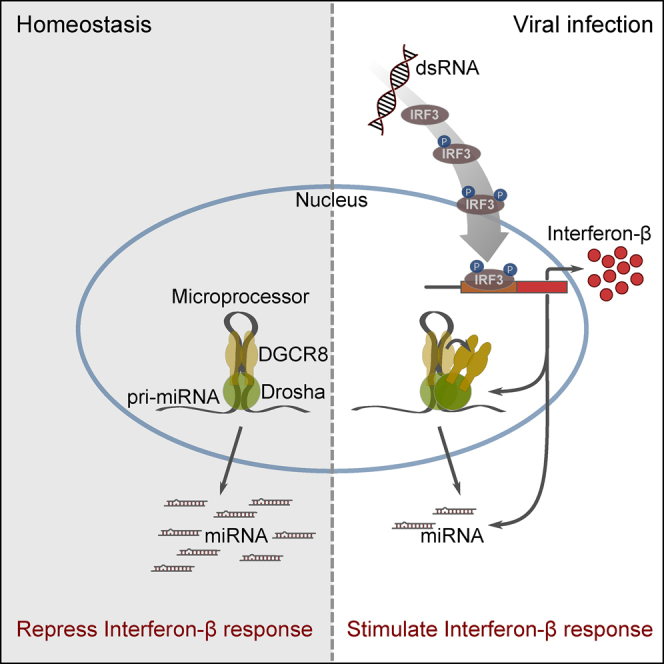

## Teaser

In order to survive viral infections, cells activate the expression of antiviral cytokines such as IFN-β. Witteveldt et al. show that this response alters the production of miRNAs by regulating the Microprocessor complex and that this regulation is necessary for the robust production of IFN-β.

## Introduction

Type I interferons (IFNs) are one of the most important classes of cytokines in the innate immune response to viral infections. Their expression is activated upon recognition of pathogen-associated molecular patterns (PAMPs) by the host pathogen recognition receptors (PRRs). Typical viral PAMPs are the viral RNA or DNA genome and, more important, double-stranded RNA (dsRNA), formed during viral replication. Two ubiquitously expressed intracellular RNA helicases from the RIG-I-like receptor family, MDA5 and RIG-I, act as PRRs for dsRNAs (Andrejeva et al., 2004, Yoneyama et al., 2004). Upon binding to the dsRNA, both RIG-I and MDA5 interact with the mitochondria-bound protein MAVS, which leads to the nuclear translocation of the transcription factors IRF3 and NF-κB (Kawai et al., 2005, Seth et al., 2005). The nuclear activity of IRF3 plays a major role in the activation of the *IFNB1* promoter, which is also facilitated by NF-κB activity (Lin et al., 1998, Schafer et al., 1998, Wathelet et al., 1998, Weaver et al., 1998, Yoneyama et al., 1998, Wang et al., 2010). Once IFN-β is expressed and secreted, it acts as an autocrine and paracrine factor by binding to the cell’s transmembrane type I IFN receptor. This binding activates the JAK/STAT signaling cascade, which induces the expression of a large number of genes, known as IFN-stimulated genes (ISGs), that are necessary for the establishment of the antiviral state, which is a crucial early line of defense against viral infections (Uzé et al., 1990, and reviewed in Stark and Darnell, 2012).

Regulation of IFN-β production is essential for cell homeostasis, as deregulation of expression can lead to apoptosis, inflammation, and immunological disorders (reviewed in Malireddi and Kanneganti, 2013). To ensure correct levels of IFN-β, it is both regulated at the transcriptional and post-transcriptional levels (Friedman et al., 1984). Although the mechanisms of its transcriptional regulation are well described, the post-transcriptional control of *IFNB1* expression remains to be completely elucidated. So far, the 3′UTR of *IFNΒ1* mRNA has been shown to be important for translational regulation, and specifically the presence of AU-rich elements (AREs) is crucial for the downregulation of this transcript, as it is only transiently expressed during infections (reviewed in Savan, 2014, Khabar and Young, 2007). In addition, microRNAs (miRNAs) act as critical regulators of IFNs and ISGs (reviewed in Sedger, 2013, Forster et al., 2015).

MiRNAs are transcribed as long precursors termed primary miRNAs (pri-miRNAs), which fold into hairpin structures that are recognized by the Microprocessor complex in the nucleus. This complex consists of the essential factors DGCR8, a dsRNA-binding protein, and Drosha, an RNase III endonuclease that cleaves pri-miRNAs at the base of the hairpin to release 50–70-nt-long pre-miRNAs. These hairpins are subsequently exported to the cytoplasm to be further processed by Dicer to form mature miRNAs (Lee et al., 2003, Landthaler et al., 2004, Gregory et al., 2004, Bernstein et al., 2001). Both Microprocessor- and Dicer-mediated processing steps are heavily regulated by additional protein factors and particular sequences contained within the precursor miRNA (reviewed in Ha and Kim, 2014).

Here we show that the activation of the IFN response extensively remodels the expression of miRNAs by influencing their biogenesis. Specifically, IFN activation impairs the first step of miRNA biogenesis by regulating Microprocessor complex activity and reducing substrate affinity. Microprocessor function can be restored by overexpressing both DGCR8 and Drosha components, suggesting that they become limiting factors during the IFN response. In our model, the transient inhibition of Microprocessor activity is essential for the induction of a robust expression of both IFN-β and ISGs and as a consequence the antiviral response.

## Results

### Activation of the IFN Response Impairs Microprocessor Activity

**Figure 1:**
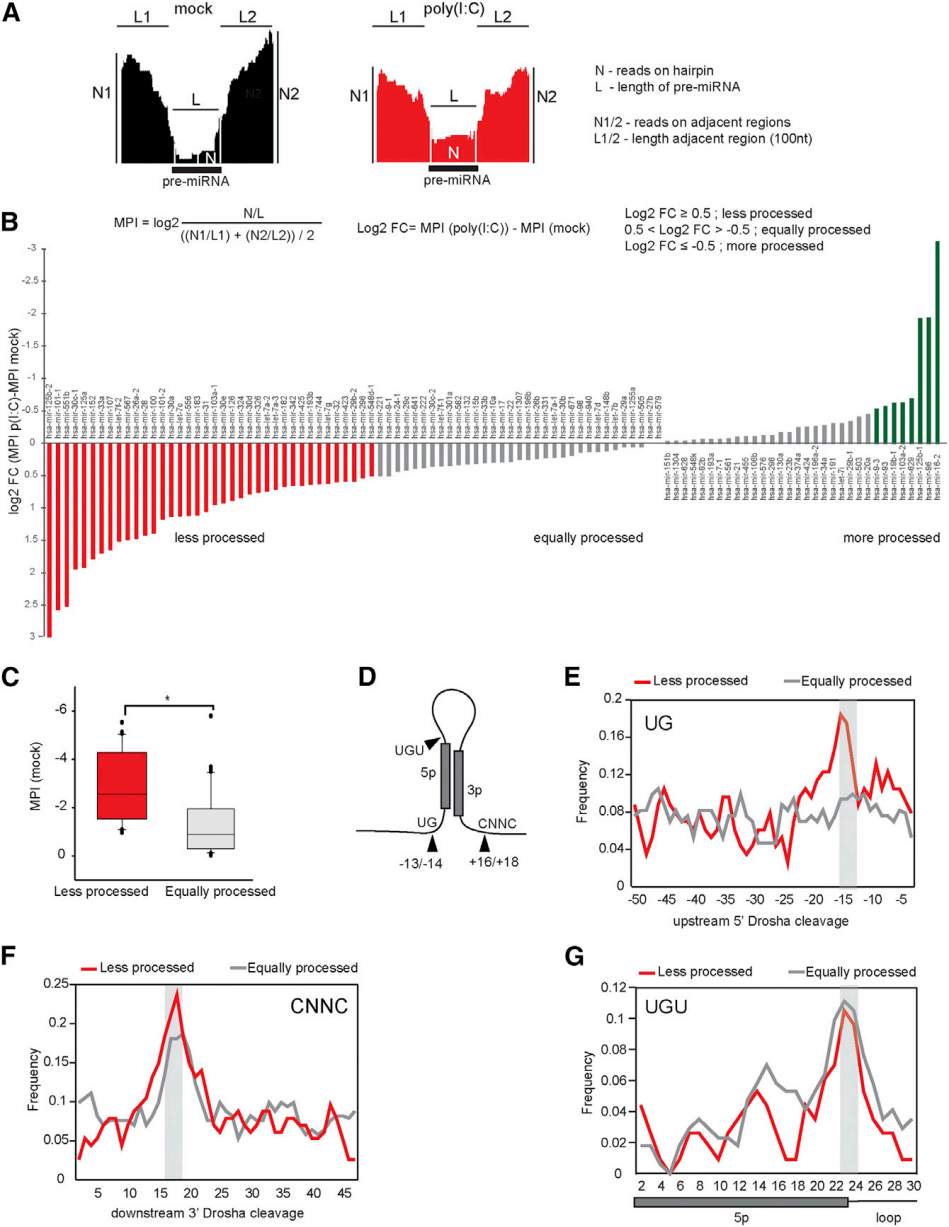
High-Throughput Analysis of pri-miRNA Processing during the IFN Response (A) For each pri-miRNA in mock-transfected cells (left) and poly(I:C)-transfected cells (right), the Microprocessor processing index (MPI) is calculated as shown. Log_2_ fold change MPI (log_2_FC) is obtained by subtraction of MPI (mock) from MPI (poly[I:C]). (B) Less processed pri-miRNAs during the IFN response result in a positive log_2_FC MPI, in red. Equally processed pri-miRNAs in gray and more processed pri-miRNAs in green. (C) Representation of the average MPI value in mock conditions for “less processed” in red and “equally processed” pri-miRNAs in gray; ^∗^p ≤ 0.05. (D) Summary of known pri-miRNA determinants of optimal Microprocessor substrates. Numbers indicate location in respect to 5′/3′ Drosha cleavage sites. (E–G) Frequency of UG (E), CNNC (F), and UGU (G) motifs for “less processed” pri-miRNAs, in red, and “equally processed,” in gray. See also Figure S1 and Table S1.

To globally assess the impact of the IFN response on the early steps of miRNA biogenesis, we performed high-throughput sequencing of RNA associated with chromatin, which has been previously shown to be enriched for pri-miRNA sequences (Conrad et al., 2014). The dsRNA analog poly(I:C) was transfected into HeLa cells to activate the IFN response, and a direct comparison of pri-miRNA cleavage between mock-treated and IFN-activated HeLa cells was made (Figures 1A and S1) (see Experimental Procedures for selection of Microprocessor-dependent miRNAs). For easy comparison, the Microprocessor processing index (MPI) of each pri-miRNA was calculated. This index takes into account the changes in the expression level of the pri-miRNA (Figure 1A, as N1 and N2), as well as the read density in the pre-miRNA region that is excised by the Microprocessor activity (Figure 1A, as N). In this way, processed pri-miRNAs have a negative MPI, and values closer to zero indicate the absence of processing. Next, the MPI of every mock-treated pri-miRNA was subtracted from the corresponding MPI of poly(I:C)-transfected cells, which represents the log_2_ fold change (difference [log_2_FC]) between the two conditions. Positive values correspond to loss of miRNA processing following poly(I:C) treatment, whereas negative values correspond to increased processing, and values close to zero indicate no changes in processing (Figure 1A). We found 38 pri-miRNAs with less processing following activation of the IFN response (log_2_FC MPI ≥ 0.5, in red), 57 similarly processed (log_2_FC MPI between −0.5 and 0.5, in gray), and only 8 pri-miRNAs that were more efficiently processed (log_2_FC MPI ≤ −0.5, in green) (Figure 1B; Table S1). The majority of the pri-miRNAs affected by the IFN response produce miRNAs that have been implicated in the regulation of the innate immune response or directly control the levels of *IFNΒ1* mRNA (Witwer et al., 2010) (for a complete list, see Figure S1D). Interestingly, these pri-miRNAs were also significantly more processed in control cells compared with pri-miRNAs whose processing did not change during the IFN response (Figure 1C). We next studied the presence of certain RNA sequence motifs to explain the difference we observed between the two groups of pri-miRNAs (less processed and equally processed). Differential analysis using the MEME suite (Bailey et al., 2009) did not yield any significant novel motifs. However, a number of previously described motifs were found (Figure 1D). The UG motif in position −14/−13 nt (upstream of the 5′ end Drosha cleavage), UGU motif at the boundary of the 5p miRNA and the terminal loop, and the CNNC motif in position +16/+18 nt (after the 3′ end Drosha cleavage site) had been previously reported to be hallmarks of Microprocessor-mediated pri-miRNA recognition (Auyeung et al., 2013). Our analyses showed that the UG motif was exclusively enriched for pri-miRNAs that are less processed during the IFN response, whereas the CNNC and UGU motif were similarly enriched (Figures 1E–1G).

These results support previous studies in which changes in processing efficiencies were assessed in the absence of the Microprocessor component Drosha (Conrad et al., 2014). Pri-miRNAs with high processing levels showed greater response to Drosha depletion compared with less processed pri-miRNAs but also increased enrichment for the CNNC, GNNU, and UG motifs. Our results suggest that IFN activation predominantly affects pri-miRNAs that are optimal Microprocessor substrates.

### IFN-Mediated Microprocessor Inhibition Is Rapid and Transient

**Figure 2:**
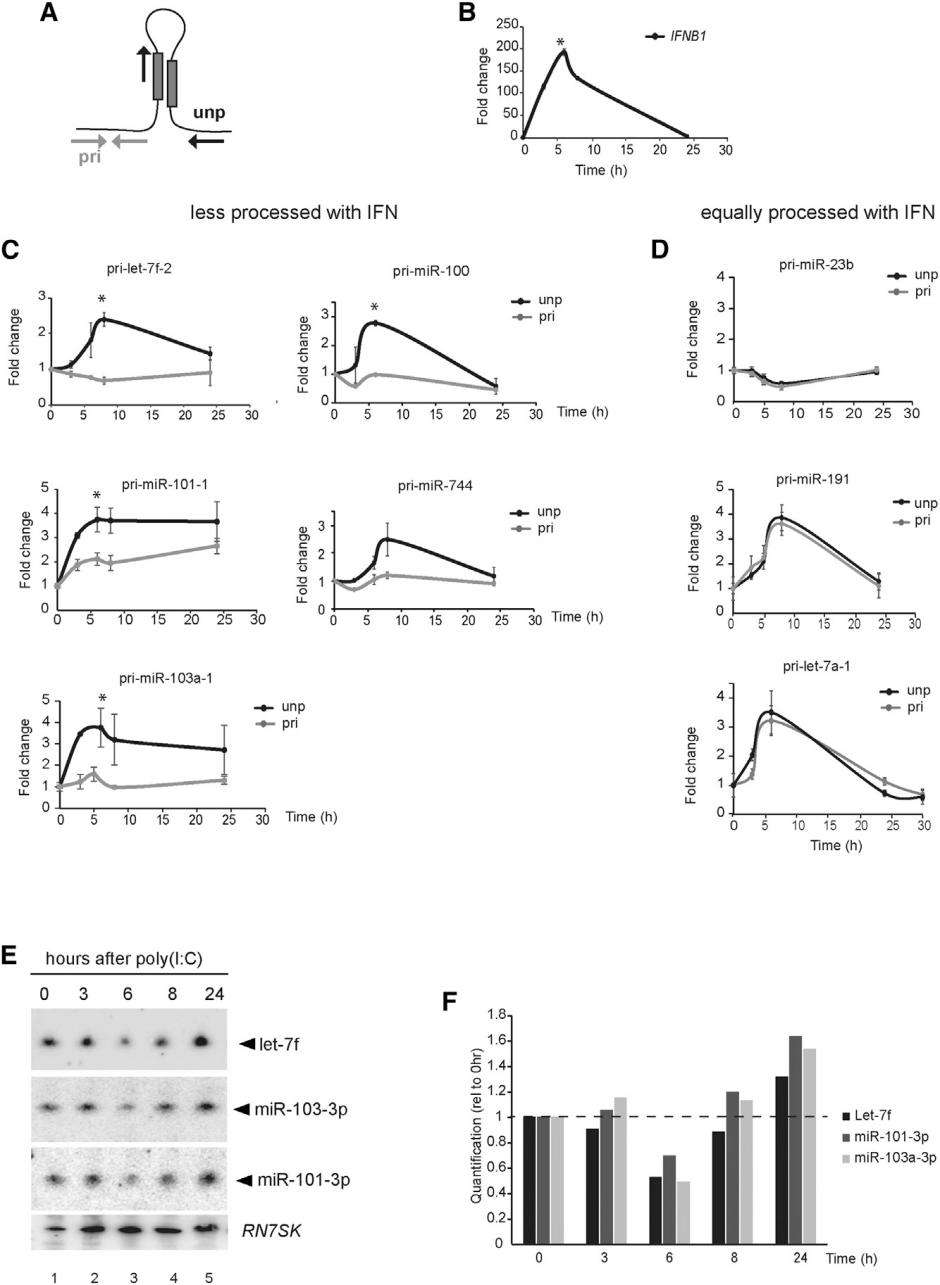
Microprocessor Processing Dynamics during the Activation of IFN Response (A) Differential qPCR method to quantify relative changes of the pri-miRNA transcripts (gray arrows) versus unprocessed pri-miRNAs levels (black arrows). (B–D) Time course analysis of *IFNB1* expression (B) and unprocessed pri-miRNAs (black, “unp”) and host transcript levels (gray, “pri”) after poly(I:C) transfection, for “less processed” (C) and equally processed pri-miRNAs (D). All graphs show the average values (n ≥ 2, biological replicates) at any time point (±SEM); ^∗^p ≤ 0.05 compared with mock. All values are normalized to *RN7SK* and expressed relative to mock (0 hr) sample. (E) Northern blot analyses, from top to bottom, let-7f, miR-103-3p, and miR-101-3p mature miRNA levels during poly(I:C) time course as in (B). (F) Quantification of mature miRNA depletion levels from (E) shown as a relative value to zero time point. See also Figures S2 and S3.

We studied the temporal dynamics of Microprocessor regulation during the IFN-β response. Cells were transfected with poly(I:C) and collected at different time points for quantification of both unprocessed pri-miRNA and transcript levels to ensure that accumulation of unprocessed pri-miRNAs is not due to changes in the transcription rate of the host transcript (Figure 2A). All five candidates from the “less processed” group consistently showed accumulation of unprocessed pri-miRNAs 4–6 hr after poly(I:C) transfection, whereas the levels of the host transcript remained constant, at the levels of both total RNA (Figure 2C) and RNA associated to chromatin (Figures S2A and S2B). Interestingly, the peak of pri-miRNA accumulation coincides with the maximum production of *IFNB1* mRNA (Figure 2B). The selected pri-miRNAs from the “equally processed” group followed different patterns. Pri-miR-23b did not significantly accumulate unprocessed pri-miRNA upon IFN-β activation, whereas pri-miR-191 and pri-let-7a-1 did show accumulation of unprocessed miRNAs. However, this coincided with increased host transcript levels, suggesting that the increase in the unprocessed levels are a consequence of increased transcription during the IFN response and not specific downregulation of these pri-miRNAs’ processing (Figures 2D and S2C).

To further investigate the functional consequences of pri-miRNA processing and the IFN-β response, we measured the expression of the mature miRNAs produced by these transcripts. Three miRNAs were selected (let-7f, miR-103-3p, and miR-101-3p) and assayed using northern blot and showed that 6 hr after poly(I:C) transfection, there was a decrease in mature miRNA levels (Figures 2E and 2F for northern blot quantification), which coincides with the maximum accumulation of unprocessed pri-miRNAs (Figure 2C). For pri-miRNAs that do not change the net ratio of processing, the levels remained constant (Figure S3B, miR-191). To test whether the decrease in mature miRNAs was also due to IFN-induced turnover, we compared mature miRNA levels by northern blot in a poly(I:C) time course in the presence of the transcription inhibitor actinomycin D (ActD). The treatment with ActD stopped the accumulation of unprocessed pri-miRNAs (Figure S3A) and the decrease in mature miRNA levels at the early time points (Figure S3B). In addition, ActD blocked *IFNB1* expression (Figure S3A), thereby preventing accurate measurements of miRNA half-life during the IFN response. These results suggest that IFN-β expression or the transcriptional program induced by poly(I:C) are essential to regulate Microprocessor activity and to observe a concomitant decrease of mature miRNA levels, which can be acting in concert with IFN-mediated miRNA degradation.

### IRF3 Activity Is Essential for Microprocessor Regulation

**Figure 3:**
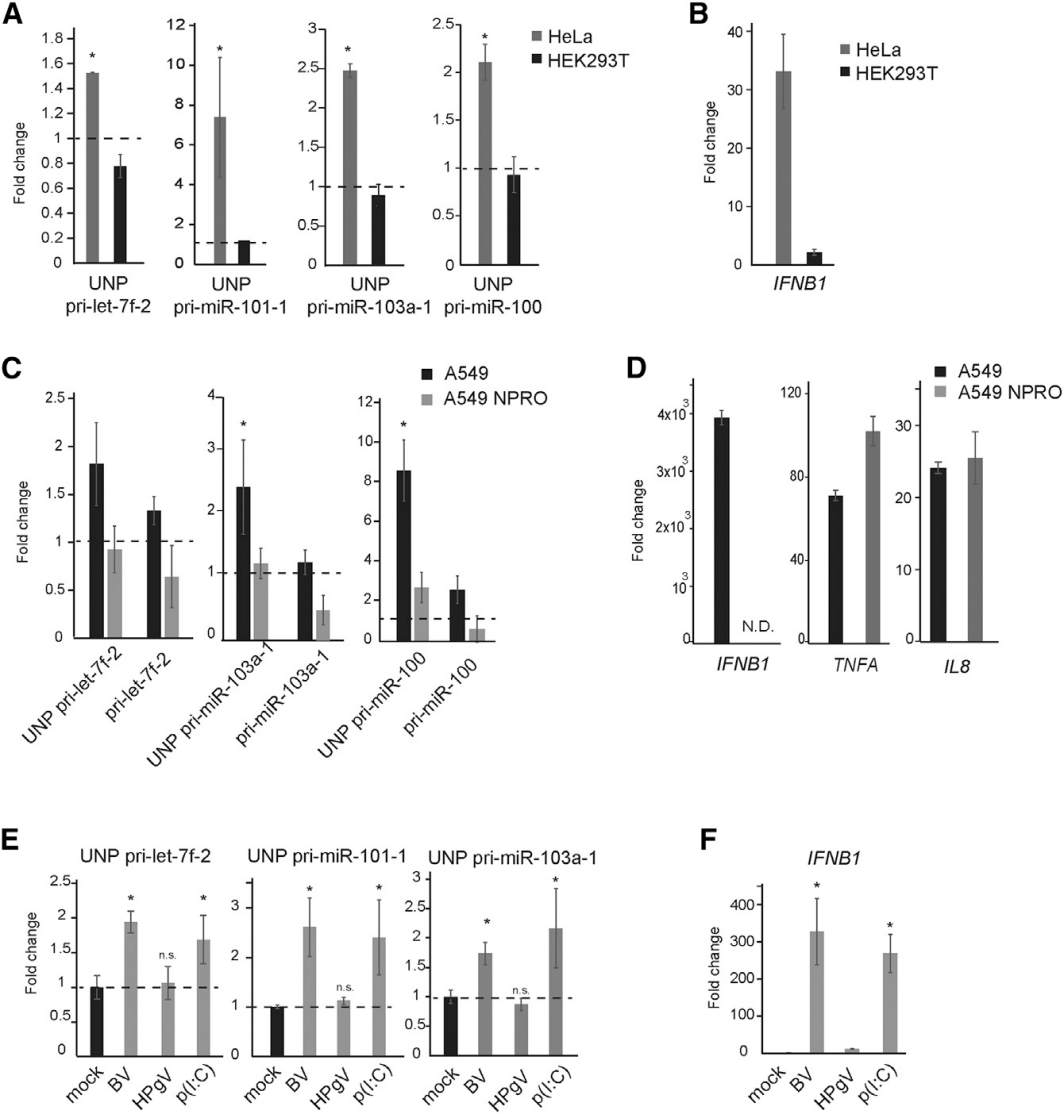
IRF3 Activity Is Essential for Regulation of Microprocessor Activity (A) Quantification of unprocessed pri-miRNAs 4 hr after poly(I:C) transfection in HeLa cells (gray) and HEK293Ts (black). Data shown are the average (n = 6, biological replicates) ± SEM; ^∗^p ≤ 0.05 compared with mock. All values are normalized to *RN7SK* and expressed relative to each mock sample (set to 1). (B) Levels of *IFNB1* mRNA induction were measured from samples in (A). (C) Quantification of unprocessed pri-miRNAs (“UNP-pri”) and host transcripts (“pri”) in wild-type (WT) A549 cell line (black) and A549-NPro (gray) 6 hr after poly(I:C) transfection. Data shown represent the average (n = 6, biological replicates) ± SEM, ^∗^p ≤ 0.05 compared with mock. All values are normalized to *RN7SK* and expressed relative to each mock sample (set to 1). (D) Quantification of *IFNB1*, *TNFA*, and *IL8* mRNA 6 hr after poly(I:C) transfection in A549 and A549-Npro cells. (E) HeLa cells were transfected with the viral-derived RNAs BV and HPgV, and poly(I:C) as a positive control. Accumulation of unprocessed pri-miRNAs was measured 4 hr post-transfection by qPCR. Values shown are average (n = 4, biological replicates) ± SEM; ^∗^p ≤ 0.05 compared with mock. All values are normalized to *RN7SK* and expressed relative to mock sample. (F) Levels of *IFNΒ1* mRNA expression were measured by qPCR for the transfected RNAs used in (E). See also Figure S3.

To confirm that *IFNB1* expression is essential for Microprocessor regulation, we compared pri-miRNA processing in HeLa and HEK293T cells. HEK293T are known to mount a poor IFN-β response to poly(I:C) because of low levels of expression of the MDA5 PRR (Rice et al., 2014). We confirmed that these cells were unable to activate *IFNB1* mRNA expression upon poly(I:C) transfection, and this correlated with their inability to regulate Microprocessor function, as no accumulation of unprocessed pri-miRNAs was observed (Figures 3A and 3B).

In another approach, we used A549 cells that are proficient in activating the expression of type I IFN and compared these with A549 cells expressing the viral-derived N Protein (NPro), which induces the degradation of the transcription factor IRF3, and consequently are unable to transcribe the *IFNB1* gene and mount an IFN response (Hilton et al., 2006). Both cell lines were transfected with poly(I:C) and accumulation of unprocessed pri-miRNAs was measured 4 hr post-transfection. Only wild-type A549 cells accumulated unprocessed pri-miRNAs (pri-let-7f-2, pri-miR-103a, and pri-miR-100) upon poly(I:C) transfection, whereas NPro cells did not show a downregulation of Microprocessor activity (Figures 3C and 3D). Importantly, NPro cells are still able to activate the transcriptional program driven by NF-κB, as *TNFA* and *IL8* mRNA expression is still induced upon poly(I:C) transfection (Figure 3D).

We next asked if viral-derived immunogenic RNAs could have the same effect on Microprocessor function and confirm that this is IFN-β response dependent and not a poly(I:C) artifact. For this purpose, we used two 4,000-nt-long viral-derived single-stranded RNAs that differ greatly in their predicted secondary structure and ability to elicit an IFN-β response (Witteveldt et al., 2014). The viral RNAs were produced by *in vitro* transcription in the absence of a cap and transfected into HeLa cells in similar amounts to poly(I:C). Only those RNAs proficient in eliciting an IFN-β response (BV and poly[I:C]) impaired Microprocessor function, showing accumulation of the unprocessed products of pri-let-7f-2, pri-miR-101-1, and pri-miR-103a (Figures 3E and 3F).

We wondered if the regulation of miRNA biogenesis is limited to cells that are activated by dsRNA or whether the paracrine action of secreted IFN-β is also able to induce this regulation. For this we added media containing type I IFN to HeLa cells and observed induction of ISGs, such as *MDA5* and *IFIT1*, but no alteration of pri-miRNA processing. These results suggest that Microprocessor regulation is associated with the activation and expression of type I IFN but not ISGs (Figures S3C and S3D). All these together suggest that an active IRF3 pathway and expression of *IFNΒ1* mRNA are essential for modulating Microprocessor complex activity during the IFN response.

### Altered Microprocessor Binding and Cleavage during IFN Activation

**Figure 4:**
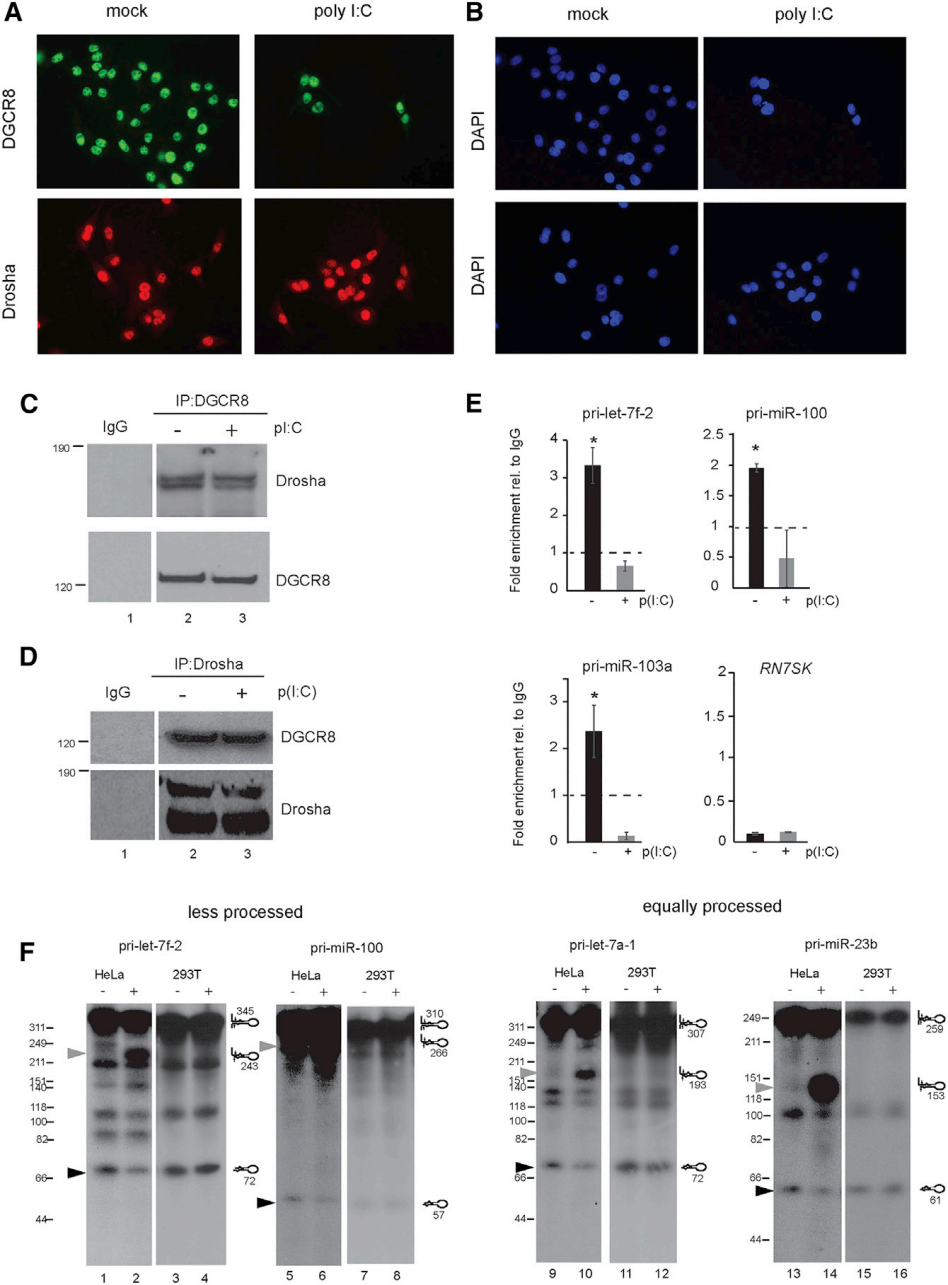
Alteration of Microprocessor Binding to pri-miRNA during the IFN Response (A) Representative immunofluorescence imaging of endogenous DGCR8 (top) and Drosha (bottom) in mock (left) and 4 hr post-poly(I:C) transfection (right) in HeLa cells. (B) DAPI staining for images in (A). (C) Co-immunoprecipitation of Drosha with DGCR8 from mock-transfected cells (lane 2) and poly(I:C)-transfected cells (lane 3). IgG serves as a immunoprecipitation negative control (lane 1). (D) Reverse co-immunoprecipitation as in (C). (E) Quantification of co-immunoprecipitated pri-miRNA with endogenous DGCR8 in normal cells (black) and poly(I:C)-transfected cells (gray). Data shown are the average of at least two experiments ± SEM; ^∗^p ≤ 0.05 when ± poly(I:C) samples are compared and relative to IgG control immunoprecipitation, set to 1 (dashed line). *RN7SK* serves as negative control for DGCR8 co-immunoprecipitation. (F) *In vitro* processing assays of radiolabeled pri-miRNAs, pri-let-7f-2 (lanes 1–4), pri-miR-100 (lanes 5–8), pri-let-7a-1 (lanes 9–12), and pri-miR-23b (lanes 13–16) with mock-transfected HeLa cell extracts (lanes 1, 5, 9, and 13), and poly(I:C)-transfected (lanes 2, 6, 10, and 14), and HEK293T mock (lanes 3, 7, 11, and 15), and poly(I:C)-transfected (lanes 4, 8, 12, and 16) cell extracts. Black arrows indicate processed pre-miRNAs, and gray arrows indicate processing intermediates. See also Figures S4 and S5.

To study the mechanism by which the activation of the IFN response modulates Microprocessor function, we assessed DGCR8 and Drosha localization after poly(I:C) transfection by immunofluorescence. Both Drosha and DGCR8 are mostly nuclear proteins and do not significantly change their localization in the presence of poly(I:C) (Figures 4A, 4B, and S4A). The weak cytoplasmic signal for Drosha has been previously reported for specific alternatively spliced Drosha isoforms (Link et al., 2016). Using labeled poly(I:C), we found that transfected poly(I:C) is mainly cytoplasmic and in the form of granules, precluding a sequestering effect of poly(I:C) on Drosha and DGCR8 in the nucleus (Figure S4B).

The integrity of the Microprocessor complex during the IFN response was measured by co-immunoprecipitating Drosha with DGCR8 in the presence or absence of an IFN response. There were no differences in the interaction between DGCR8 and Drosha, as similar amounts of co-immunoprecipitated Drosha were observed (Figure 4C, compare lanes 2 and 3). This was verified with the reverse co-immunoprecipitation experiment (Figure 4D). We assayed the binding of DGCR8 to pri-miRNAs during the IFN response by immunoprecipitating endogenous DGCR8 protein from mock- and poly(I:C)-transfected cells and isolating the associated RNA for qPCR quantification. All three pri-miRNAs tested showed decreases in DGCR8 binding during the IFN response (Figure 4E); in contrast, DGCR8 recovery did not change upon poly(I:C) stimulation (Figure S4D). DGCR8 was also binding less efficiently to pri-miRNAs whose net processing rates were unaffected by the IFN response (Figure S4C), suggesting that IFN activation alters DGCR8 binding ability in a non-selective manner.

We next examined if the reduced DGCR8 binding ability also has an impact on the Microprocessor processing ability *in vitro*. Microprocessor cleavage was measured for four different pri-miRNAs: two whose processing was affected during the IFN response (pri-let-7f-2 and pri-miR-100) and two unaffected (pri-let-7a-1 and pri-miR-23b). Radiolabeled pri-miRNAs were incubated with extracts from mock- or poly(I:C)-transfected HeLa and HEK293T cells and analyzed by gel electrophoresis. All pri-miRNAs showed decreases in cleaved pre-miRNA hairpins in extracts from poly(I:C)-transfected HeLa cells (Figure 4F, compare lanes 1 and 2, 5 and 6, 9 and 10, and 13 and 14; black arrows denote cleaved pre-miRNA hairpin). As expected, HEK293T-derived extracts showed similar pre-miRNA hairpin cleavage levels for all treatments (Figure 4F, compare lanes 3 and 4, 7 and 8, 11 and 12, and 15 and 16). These results support the model that Microprocessor activity regulation is dependent on the ability of the cell to mount an IFN-β response and can act on a non-selective manner on any pri-miRNA *in vitro*.

We also observed the accumulation of an unprocessed intermediate with HeLa cell extracts (Figure 4F, marked with gray arrows, and Figure S5 for shorter exposed images). The sizes of these unprocessed species match pri-miRNAs that fail to process the 5′ end arm of the pri-miRNA hairpin (Figures 4F and S5 for complete details). These results suggest that the IFN-mediated regulation of Microprocessor activity can be recapitulated *in vitro* and results in less processing efficiency independently of the identity of the pri-miRNA.

### Microprocessor Activity Regulation Is Essential for a Strong IFN Response

**Figure 5:**
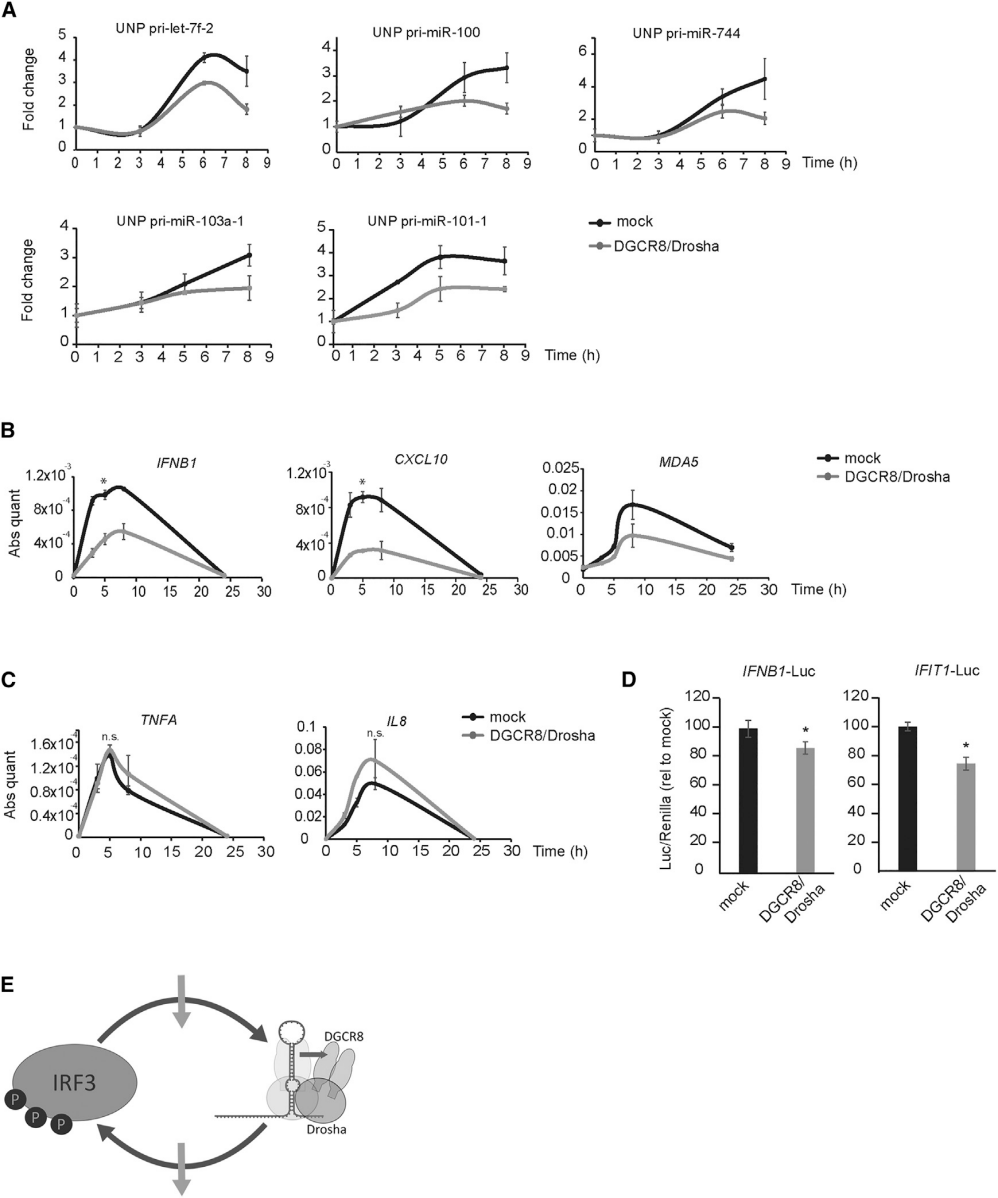
Overexpression of DGCR8 and Drosha Rescues pri-miRNA Processing Defect and Decreases the Type I Interferon Response (A) Time course of unprocessed pri-miRNA accumulation after poly(I:C) transfection in the presence of overexpressed DGCR8 and Drosha (gray) and mock-transfected cells (empty plasmids, in gray). Data shown are the average (n = 2, biological replicates) ± SEM. All values are normalized to *RN7SK* and expressed relative to mock (0 hr) sample. (B and C) Time course of *IFNB1*, *CXCL10*, and *MDA5* (B) and *TNFΑ* and *IL8* (C) mRNAs expression after poly(I:C) transfection in mock-transfected (black) and DGCR8 and Drosha overexpressing HeLa cells (gray). Data shown are the average (n = 2, biological replicates) ± SEM; ^∗^p ≤ 0.05 comparing mock and DGCR8/Drosha overexpression. All values are normalized to *RN7SK*, as in (A). (D) Luciferase activity driven by *IFNB1* promoter (left) and *IFIT1* promoter (right) after poly(I:C) transfection, in mock (black) and DGCR8 and Drosha overexpressing HeLa cells (gray). Data shown are the average (n = 3, biological replicates) ± SEM normalized to firefly Renilla values; ^∗^p ≤ 0.05 comparing mock and DGCR8/Drosha overexpression. (E) Proposed model for feedback loop regulation of Microprocessor activity during the activation of the IFN response.

The rapid and transient regulation of Microprocessor activity and change in affinity to its substrates during the IFN response suggests a direct modification of this complex that might be overcome by overexpressing both wild-type DGCR8 and Drosha. We compared the accumulation of unprocessed pri-miRNAs in normal and DGCR8/Drosha overexpressing cells after poly(I:C) stimulation. All the tested pri-miRNAs accumulated less unprocessed pri-miRNA after poly(I:C) transfection when DGCR8 and Drosha were overexpressed, suggesting that increased levels of the Microprocessor factors neutralize the regulatory effect of the IFN response (Figure 5A). This regulation of miRNA biogenesis is important for a robust IFN response as we observed a significant decrease in *IFNΒ1* mRNA when DGCR8 and Drosha are overexpressed (Figure 5B). A similar pattern was found for the ISGs *CXCL10* and *MDA5* (Figure 5B). Conversely, *TNFΑ* and *IL8* mRNAs, which are NF-κB transcription-dependent genes, were not significantly affected by DGCR8 and Drosha overexpression, suggesting that IFN-β-mediated Microprocessor regulation is essential to modulate the IRF3 transcriptional program in this cellular context (Figure 5C).

To uncouple the effects of miRNAs on the regulation of *IFNΒ1* transcription from the direct regulation of the *IFNΒ1* mRNA itself by miRNAs, we used a reporter plasmid containing the *IFNΒ1* promoter driving luciferase expression. Overexpression of the Microprocessor components led to a consistent and statistically significant (10%) reduction of luciferase activity upon poly(I:C) transfection (Figure 5D). In addition, the activity of a luciferase reporter driven by the ISG *IFIT1* promoter allows the indirect monitoring of endogenous type I IFN production, as the most prominent inducer of its expression is IFNα/β (reviewed in Fensterl and Sen, 2011). This reporter displayed a much more pronounced reduction in luciferase activity (30%) when both Microprocessor components were overexpressed, corroborating the amplifying effect of lower IFN-β induction (Figure 5D). All these experiments led us to hypothesize that the regulation of Microprocessor activity during the IFN response is essential to post-transcriptionally regulate *IFNB1* mRNA levels and, as consequence, the levels of ISGs. On the other hand, we showed that the activity of IRF3 is essential in the modulation of Microprocessor activity, which suggests a negative feedback loop between the IRF3-*IFNΒ1* transcriptional axis and Microprocessor activity (Figure 5E).

## Discussion

Host miRNAs are essential in regulating many cellular processes, including the antiviral response, where they can act as proviral or antiviral factors (reviewed in Russo and Potenza, 2011). However, viruses can also encode for viral miRNAs and use the canonical miRNA biogenesis machinery from the host to exert their functions (Grundhoff and Sullivan, 2011; reviewed in Kincaid and Sullivan, 2012). Both Drosha and Dicer have also been shown to have antiviral properties in mammalian organisms. The nuclease Drosha can cleave viral RNA transcripts, inducing their degradation after shuttling to the cytoplasm (Shapiro et al., 2014), and recent efforts have expanded this observation to other RNase III nucleases from diverse kingdoms (Aguado et al., 2017). Although more controversial, Dicer has been shown to have an antiviral role in mammals by cleaving viral transcripts to create antiviral small interfering RNAs (siRNAs), which is reminiscent of Dicer function in invertebrates and plants (Maillard et al., 2013, Li et al., 2013, Li et al., 2016). All these findings show the complex relationship between miRNAs and the antiviral response. To avoid viral-specific induced regulation of the IFN response, we decided to use a dsRNA analog to mimic the induction of the IFN response during a viral infection and study its impact on miRNA biogenesis. Our study revealed that Microprocessor complex activity is transiently inhibited during the activation of the IFN response and as a consequence leads to reduced levels of specific miRNAs. Many of these downregulated miRNAs have been shown to regulate genes involved in the innate immune response, such as pri-miR-125-a and pri-miR-125-b regulating MAVS expression but also miRNAs that directly regulate *IFNB1* mRNA levels, such as the let-7 family or miR-26a (Witwer et al., 2010, Hsu et al., 2017; Figures 1B and S1). All these results agree with the suggested role for miRNAs as negative regulators of the type I IFN response in mammals during homeostasis, in which the total absence of miRNAs by genetic ablation of *Dicer* leads to elevated levels of type I IFN-dependent genes (Ostermann et al., 2012). Intriguingly, our analysis also showed that only a few pri-miRNAs were more efficiently processed during the IFN response, such as the case for pri-miR-9. This miRNA has been shown to increase the expression of IFN regulated genes and to be upregulated by LPS exposure (Gao et al., 2013, Bazzoni et al., 2009). This implies that some miRNAs are essential for the IFN response and that they may have developed mechanisms to bypass the general inhibition of the Microprocessor activity during the activation of IFN.

The efficiency with which pri-miRNAs are processed is a major determinant of miRNA expression (Conrad et al., 2014). The processing efficiency of the Microprocessor complex is regulated by many factors, including the pri-miRNA sequence composition and additional auxiliary proteins that enhance or repress Microprocessor binding activity (Guil and Cáceres, 2007, Fernandez et al., 2017; reviewed in Connerty et al., 2015). Our results show that IFN-affected pri-miRNAs are enriched for features characteristic of optimal Microprocessor substrates. They are efficiently processed by Drosha in homeostasis (low MPI values) but also contain motifs that are hallmarks of optimal Microprocessor recognition and processing, as previously described (Auyeung et al., 2013). In addition, only IFN-regulated pri-miRNAs harbor a UG motif in position −13/−14 nt upstream of the 5′ end Drosha cleavage site, which can potentially explain the difference in the behavior of these pri-miRNAs upon IFN activation. Because of the small number of miRNAs showing increased expression upon IFN induction, no significant differences in motifs or structure could be found (Figures 1E–1G). Additional experiments will aim at identifying the specific function of the UG motif in the context of the IFN response and more specifically in driving Drosha cleavage to the 5′ end of the pre-miRNA hairpin.

Although the precise mechanism by which IFN activation leads to the impairment of Microprocessor activity is still unknown, we know that active IRF3 is crucial, and not poly(I:C) specific, as the same effect can be recapitulated with viral RNAs that also activate the IFN response (Figure 3). Immunofluorescence data argue against a sequestering effect of the poly(I:C) on the dsRNA-binding proteins DGCR8 and Drosha, as both components do not co-localize. Our current hypothesis proposes that the Microprocessor complex is post-translationally regulated during the IFN response, leading to a quick and transient decrease in miRNA levels, which allows a fast and reversible mechanism. However, miRNA stability data cannot exclude IFN-dependent turnover processes acting in concert to regulate mature miRNA levels.

Our results also show that regulation of the Microprocessor activity is essential for a robust IFN response, specifically for the IRF3 transcriptional target, *IFNΒ1*, but not for NF-κB-dependent genes, such as *TNFΑ* (Figure 5). The fact that IRF3 activity is essential for the inhibition of Microprocessor activity, and that this regulation mainly affects IRF3 transcriptional targets, implies the presence of a feedback loop. This finding further highlights the complex network of interactions acting in concert to control the expression of *IFNB1*. The levels of IFN-β expression are crucial for the effective activation of the antiviral response network, and viruses have successfully exploited this complex pathway to develop factors that act to block the production of IFN-β (reviewed in García-Sastre, 2017). However, an uncontrolled production of IFN-β also has a negative impact on the host. A group of disorders in humans, associated with elevated levels of type I IFN, are caused by a very diverse range of genetic mutations in factors that can either lead to an abnormal accumulation of endogenous nucleic acids or enhanced sensitivity of the nucleic acid receptors and signaling pathways (reviewed in Crow, 2015). Because some miRNAs are negative regulators of the type I IFN response, it will be of extreme interest to identify the key miRNAs that control the IFN response in mammalian systems and whose deregulated expression can lead to abnormal IFN expression.

## Experimental Procedures

### Cell Culture, Plasmids, and Transfections

HeLa, HEK293T, A549 and A549-NPro cells were maintained in standard cell culture conditions (DMEM containing 10% fetal calf serum [FCS] at 37°C, 5% CO_2_). Poly(I:C) (HMW, tlrl-pic; Invivogen) transfections were performed using Lipofectamine 2000 following the manufacturer’s instructions at 1 μg/mL final concentration. Plasmids containing FLAG-DGCR8 and FLAG-Drosha and luciferase and Renilla vectors were transfected in HeLa cells using Lipofectamine 2000 (as in Macias et al., 2015). RNAs derived from BV (Bunyamwera) and HPgV (Human Pegivirus) viruses were generated by *in vitro* transcription and transfected using Lipofectamine 2000 at 1 μg/mL (as in Witteveldt et al., 2014). For miRNA stability studies, ActD was added to a final concentration of 5 μg/mL for the times indicated.

### Chromatin-Associated RNA Sample Preparation and Sequencing

Chromatin-associated RNA was prepared as previously described (Conrad et al., 2014). Four mock-transfected and four poly(I:C)-transfected chromatin-associated RNA preparations were generated for strand-specific RNA transcriptome sequencing, including ribo-zero rRNA depletion and random fragmentation and strand-specific library construction and sequenced by Illumina HiSeq 4000, 100PE. Cytoplasmic, nucleoplasmic, and chromatin fractionations were validated by western blot with antibodies against tubulin (CP06; Millipore) and histone H3 (4499; Cell Signaling).

### Analysis of Chromatin-Associated RNA Libraries

Raw fastq-format sequences were quality assessed using FASTQC (https://www.bioinformatics.babraham.ac.uk/projects/fastqc/). On the basis of the output of the FASTQC analysis, the raw fastq sequences required no further pre-processing to remove contaminating primers. Pre-miRNAs and their mature sequences were downloaded from mirBase as an Excel file (ftp://mirbase.org/pub/mirbase/21/miRNA.xls.zip). Hairpin regions were extracted from the pre-miRNAs by removing, where possible, all sequence upstream of the 5′ end of the designated 5p mature sequence and all sequence downstream of the 3′ end of the designated 3p mature sequence (Table S2). Mapping co-ordinates on the human genome were obtained when the hairpins were aligned using bowtie2 (version 2.2.7, parameters: --very-sensitive -p 6 --no-unal; http://bowtie-bio.sourceforge.net/bowtie2/manual.shtml) to the human reference genome (hg19.p4) and subsequently sorted and indexed using samtools (version 1.3; http://www.htslib.org). Motifs were searched within the precursor and their flanking regions using the command-line version of EMBOSS version 6.6.0.0 fuzznucc (http://emboss.bioinformatics.nl/cgi-bin/emboss/fuzznuc). The weighted frequencies of motifs were calculated using the SSE package (www.virus-evolution.org) with a sliding window of 3. A “bedfile” of miRNA precursor mapping locations was generated from the bowtie2 BAM file outputs. RNA-derived sequences were aligned as single ends to the human reference genome (hg19.p4) or predicted transcripts set (Ensembl, “Rel83,” Release83 via BioMart; http://www.ensembl.org) using bowtie2 (version 2.2.7; using parameters --very-sensitive-local --no-unal). For differential expression analyses, counts and read depths were derived for transcripts, and miRNA precursor regions (including flanking sequences as appropriate) were extracted using samtools (version 1.3) and/or bedtools (version 2.23.0; http://bedtools.readthedocs.io/en/latest/). Groupwise comparisons, plots, and further processing were done using Bioconductor (http://bioconductor.org) packages within the R environment. Raw counts for each Rel83 transcript identified by one or more reads were obtained from the BAM-format alignment data using bedtools. Transcript counts for each sample were scaled to the lowest sample count total, converted to log_2_, and quantile-normalized prior to groupwise comparison (dsRNA-treated relative to mock) using linear modeling (limma package in Bioconductor; Ritchie et al., 2015). MPI values were generated as described previously (Conrad et al., 2014) and as shown in Figure 1A. The “maximum” regional read depth measures were used for further analyses. Ratios (log_2_) for reads aligning were calculated for the shoulder regions (100 bases, fixed) relative to those aligning to the known precursor region. The log_2_FC MPI was calculated as the difference between dsRNA and mock MPI values. In order to focus on relevant pri-miRNAs, the following criteria were applied: (1) a minimal pri-miRNA expression of at least a maximum of 30 reads on each side of the hairpin; (2) discard annotated miRNAs that are synthesized independently of the Microprocessor (annotated mirtrons; Ladewig et al., 2012) or do not bind to DGCR8 Microprocessor component, as identified by DGCR8 HITS-CLIP (Macias et al., 2012); (3) and are being cleaved by the Microprocessor in mock conditions (MPI mock < 0). After calculating the log_2_FC MPI, only candidates with changes ≥0.5 (less processed during IFN), ≤−0.5 (more processed), and ±0.5 (equally processed) were kept for further analyses. For a complete list of selected miRNAs, see Table S1.

### RNA Extraction and qRT-PCR Analysis

Total RNA was extracted using Trizol or Trizol LS following the manufacturer’s instructions and used to synthesize cDNA using Transcriptor Universal cDNA Master (Roche). qPCR was carried out with LightCycler 480 SYBR Green I Master mix (Roche) in a LightCycler 480 Instrument. Oligonucleotides used are listed in Table S3.

### Northern Blot for miRNAs

Total RNA (15 μg) was loaded on a 10% TBE-urea gel and transferred on a positively charged nylon membrane for 1 hr at 250 mA. After UV crosslinking, the membrane was pre-hybridized for 4 hr at 40°C in 1× saline sodium citrate (SSC), 1% SDS (w/v), and 100 mg/mL single-stranded DNA (ssDNA; Sigma-Aldrich). Radioactively labeled probes corresponding to mature let-7f, miR-103-3p, and miR-101-3p were synthesized using the mirVana miRNA Probe Construction Kit (Ambion) and hybridized overnight in 1× SSC, 1% SDS (w/v), and 100 mg/mL ssDNA. After hybridization, membranes were washed four times at 40°C in 0.2× SSC and 0.2% SDS (w/v) for 30 min each. Blots were analyzed using a PhosphorImager (Molecular Dynamics) and ImageQuant TL software for quantification. Oligonucleotides used are listed in Table S3.

### Immunoprecipitations and Association to pri-miRNAs

Endogenous DGCR8 was immunoprecipitated from a 10 cm plate of HeLa cells with 1 μg of antibody (ab90579) coupled to Protein A Magnetic Beads (88845; Pierce) in IP buffer (50 mM Trsi [pH 7.5], 150 mM NaCl, 1 mM EDTA, 1% Triton X-100, 200 U RNasin, and protease inhibitor cocktail). After overnight binding, beads were washed five times for 5 min at room temperature with IP buffer (200 mM NaCl). For analysis of co-immunoprecipitated pri-miRNAs, RNA was extracted from beads, as well as from input samples, using Trizol LS. Samples were consequently treated with DNase I for 15 min at 37°C, and the RNA was extracted by phenol/chloroform and ethanol precipitation. Input and immunoprecipitated RNA were quantified using Transcriptor Universal cDNA Master (Roche) followed by qPCR amplification with Light Cycler 480 SYBR Green I Master. The amount of immunoprecipitated RNA was normalized to the input fraction and was expressed relative to the negative control (IgG, set arbitrarily to 1). Oligonucleotides used are listed in Table S3. For western blot analyses, beads after immunoprecipitation were boiled, and eluates were loaded in 4%–12% Bis-Tris gels, transferred to nitrocellulose membranes, and hybridized with antibodies against DGCR8 and Drosha. For detection, a secondary antibody couple to horseradish peroxidase (HRP) that recognizes only the non-denatured form of IgG was used (ab131666).

### Immunofluorescence

HeLa cells were grown on coverslips, fixed with 4% paraformaldehyde, and permeabilized with 0.1% Triton X-100 in PBS. Cells were blocked in PBG (1% BSA, 0.01% Triton X-100 in PBS) for 1 hr at room temperature. After blocking, cells were incubated with primary antibodies against DGCR8 or Drosha (ab90579 and NBP1-03349, respectively) followed by anti-rabbit Alexa Fluor 488 antibodies (A11070), both diluted in PBG buffer. Coverslips were washed three times in PBS containing 0.01% Triton X-100 and mounted in slides with DAPI containing mounting medium (VECTASHIELD, H-1200). Fluorescein-labeled poly(I:C) was used to visualize localization of transfected poly(I:C) (tlrl-pi*cf*; Invivogen). Images were processed using ImageJ software (NIH).

### *In Vitro* Processing Assays

Templates for RNA synthesis and radiolabeling of pri-miRNA substrates were obtained by PCR of human genomic DNA (see oligonucleotides in Table S3). PCR products were cloned in pGEM-T Easy Vector (A1360) and sequenced. Transcription reactions were performed with T7-polymerase in the presence of 40 μmol of ^32^-P-UTP. RNA probes were gel-purified, phenol-extracted, and ethanol-precipitated. Extracts from mock- and poly(I:C)-transfected HeLa and HEK293T cells were prepared by resuspending cells after transfection in 500 μL of buffer D (20 mM HEPES-KOH [pH 7.9], 100 mM KCl, 0.2 mM EDTA, 0.5 mM DTT, 0.2 mM PMSF, 5% [w/v] glycerol, and protease inhibitor cocktail [04693159001]), followed by sonication (five pulses, 10 s each, low amplitude). *In vitro* processing reactions were performed in 30 μL containing 50% (v/v) of cell extract, 0.5 mM ATP, 20 mM creatine phosphate, 3.2 mM MgCl_2_, and 50,000 cpm of each pri-miRNA and incubated at 37°C for 30 min. Reactions were stopped by addition of proteinase K followed by phenol-chloroform extraction and ethanol precipitation and separated in a 10% TBE-UREA gel. Gels were exposed overnight to film at −80°C.

### Luciferase Assays

HeLa cells were transfected with *IFNΒ1*-Luc and *IFIT1*-Luc and TK-Renilla as a control (kind gift from Prof. G. Towers) and co-transfected with empty plasmids or plasmids overexpressing DGCR8 and Drosha. After 48 hr, poly(I:C) was transfected using Lipofectamine 2000, and cells were lysed after 8 hr using passive lysis buffer (Promega). The levels of Firefly and Renilla luciferase were measured using the Promega Dual Luciferase Reaction system on a Varioskan Flash Plate reader.

### Statistical Methods

Unless otherwise stated, values represent mean ± SEM on the basis of at least three independent experiments. Asterisks indicate statistical significance (^∗^p < 0.05) on the basis of Student’s t test.

## Data and Software Availability

The accession number for the raw and processed sequencing data reported in this paper is GEO: GSE109260.

## Supplemental Information

Document S1. Figures S1–S5 and Tables S1 and S3

Table S2. Human Pre-miRNA Hairpins, Related to Figure 1 and Experimental Procedures List of annotated human pre-miRNA sequences.

Document S2. Article plus Supplemental Information
