# Supplementary material for: Inhibition of Microprocessor Function during the Activation of the Type I Interferon Response
Source: Cell Rep. 2018 Jun 13;23(11):3275–85. doi: 10.1016/j.celrep.2018.05.049 (PMC6019736; doi:10.1016/j.celrep.2018.05.049)
Supplement: Document S1. Figures S1–S5 and Tables S1 and S3 [file mmc1.pdf]

Cell Reports, Volume *23*

Supplemental Information

Inhibition of Microprocessor Function during the Activation of the Type I Interferon Response

Jeroen Witteveldt, Alasdair Ivens, and Sara Macias

Figure S1

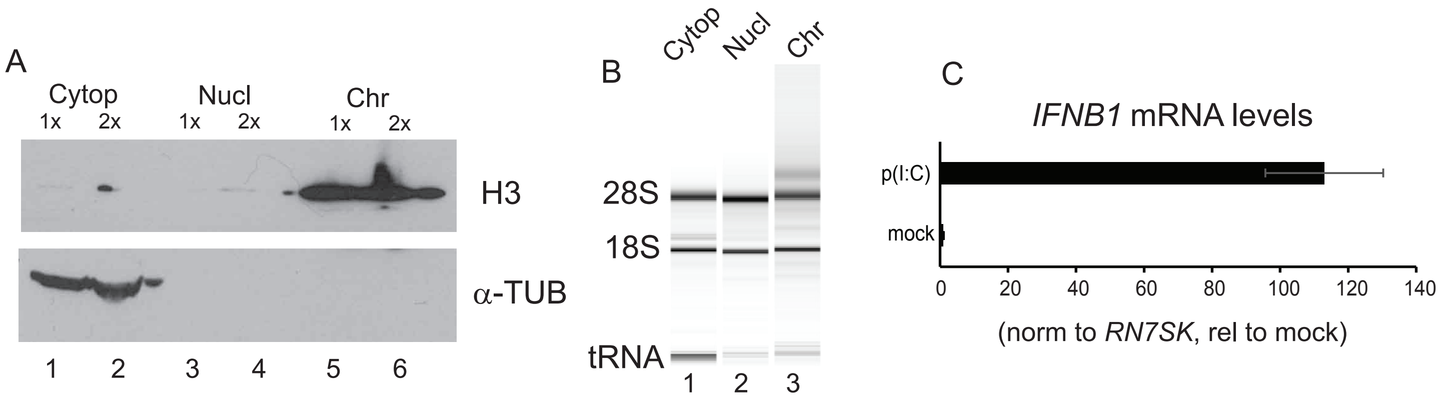

D

Locus	Targets	Reference
**hsa-mir-125b-2**	**IRF4**	Chaudhuri AA. MicroRNA 125b potentiates Macrophage activation. J Immunol 2011; 187:5062-68
	**TNFa**	Tili et al. Modulation of miR-155a nd miR-125b levels following lipopolysaccharide/TNF-alphastimulation... J Immunol 2007; 179:5082–9.
	**MAVS**	C-Y Hsu. MicroRNA-125a and -b inhibt A20 and MAVS...J Clin Invest 2017; 2(7):e90443
**hsa-mir-30c-1**	**JAK1**	Zhang Q. MicroRNA-30c is utilized by PRRSV in immune evasion thorugh targeting JAK-1 in the type-I interferon signaling pathway. J Immunol 2015;194 Suppl 1
**hsa-mir-125a**	**MAVS**	C-Y Hsu. MicroRNA-125a and -b inhibt A20 and MAVS...J Clin Invest 2017; 2(7):e90443
**hsa-mir-152**	**TLR signaling**	Liu et al. MicroRNA-148/152 Impair Innate Response and Antigen Presentation of TLR-Triggered Dendritic Cells… J Immunol 2010; doi: 10.4049/jimmunol.1001573
**hsa-mir-33a**	**TLR signaling**	Lai L. MicroRNA-33 regulates the innate immune response via ATP-binding cassette transporter-mediated... JBC 2016; doi:10.1074/jbc.M116.723056
**hsa-mir-107**	**TLR signaling**	Xue et al. Downregulation of microRNA-107 in intestinal CD11c+ myeloid cells in response…Eur J Immunol 2014; 44: 673–682
**hsa-let-7f-2**	**IL23R**	Li et al. IL-23 Receptor Regulation by Let-7f in Human CD4+ Memory T Cells. J Immunol 2011; doi: 10.4049/jimmunol.1000917
**hsa-mir-26a-2**	**IFNB**	Witwer KW. MicroRNA regulation of IFN-b protein expression: rapid and sensitive modulation of the innate immune responses. J Immunol 2010;184(5):2369-76
**hsa-mir-100**	**down by IFN**	Forster SC. MicroRNA as type I Interferon-regulated transcripts and modulators of the innate immune response. Front Immunol 2015; 6:334
**hsa-mir-30a**	**SAMHD1**	Riess M. Interferons Induce Expression of SAMHD1 in Monocytes through Downregulation of miR-181a and miR-30a. Journal of Biol Chem 2016; doi:10.1074/jbc.M116.752584
**hsa-let-7c**	**IL10**	Jiang et al. Altered let-7 expression in Myasthenia gravis and let-7c mediated regulation of IL-10 by directly targeting IL-10…Int Immunopharm 2012; 14:217-223
**hsa-mir-31**	**IL25/IRF1/IFNA2**	Shi et al. The signaling axis of microRNA-31/interleukin-25 regulates Th1/Th17-mediated inflammation…Mucosal Immunol 2017; 10:983–995
		Moffet HF. The microRNA miR-31 inhibits CD8+ T cell function in chronic viral infection. Nat Immunol 2017. doi:10.1038/ni.3755
**hsa-mir-30e**	**down by IFN**	Wang et al. Identification of Resting and Type I IFN-Activated Human NK Cell miRNomes Reveals MicroRNA-378 and MicroRNA-30e…J Immunol 2012; 189:211-221
**hsa-mir-126**	**innate immunity**	Ferretti et al. miR-126, a new modulator of innate immunity. Cell Mol Immunol 2014; 11(3): 215–217.
**hsa-let-7a-2**	**STAT3**	Hu et al. Let-7a Inhibits T-Cell Proliferation and IFN-γ Secretion by Down-Regulating STAT3 Expression...Cell Physiol Biochem 2017;42:115–125
**hsa-let-7a-3**	**STAT3**	
**hsa-mir-342**	**antiviral**	Robertson et al. An Interferon Regulated MicroRNA Provides Broad Cell-Intrinsic Antiviral Immunity through…PLOS Biol 2016; 14(3): e1002364
**hsa-mir-744**	**IFN**	Zhang X. miR-744 enhances type-I interferon signaling pathway by atrgeting PTP1B in primary human renal mesangial celss. Scientific Reports 2015. doi:10.1038/srep12987
**hsa-let-7g**	**IL6**	Huang et al. MicroRNA-142-3p and let-7g Negatively Regulates Augmented IL-6 Production...Int J Biol Sci 2017; 13(6):690-700
**hsa-mir-32**	**IL1b/IL6/TNFa**	Zhang et al. TLR-4/miRNA-32-5p/FSTL1 signaling regulates mycobacterial survival and inflammatory responses…Exp Cell Res 2017; 352:313-321
**hsa-mir-29b-2**	**innate immunity**	Ma et al. The microRNA miR-29 controls innate and adaptive immune responses…Nat Immunol 2011; doi:10.1038/ni.2073
**hsa-mir-296**	**antiviral**	Pedersen et al. Interferon modulation of cellular microRNAs as an antiviral mechanism. Nature 2007; doi:10.1038/nature06205
**hsa-mir-548d-1**	**IFNλ**	Li et al. MicroRNA-548 down-regulates host antiviral response via direct targeting of IFN-λ1. Prot Cell 2013; DOI 10.1007/s13238-012-2081-y

Figure S1. Pri-miRNAs affected by IFN produce miRNAs controlling innate immunity. Related to Figure 1 (**a**) Western blot analysis of cytoplasmic (cytop), nucleoplasmic (nucl) and chromatin (chr) fractions. Histone 3 (H3) serves as a chromatin marker, and α-tubulin (α-tub) as a cytoplasmic marker. (**b**) Distribution of RNA species (rRNA 28S and 18S, and tRNA) by using a Bioanalyzer, in cytoplasm (1), nucleoplasmic (2) and chromatin fractions (3). (**c**) qRT-PCR analysis of *IFNB1* mRNA induction by poly(I:C) transfection from chromatin-associated RNA fractions, average (n=3, biological replicates) +/- s.e.m is represented. (**d**) List of pri-miRNAs with defective Microprocessor-mediated processing during the IFN response (Locus), followed by known targets in the IFN pathway or the innate immune response (target), including the bibliographical reference for each.

Figure S2

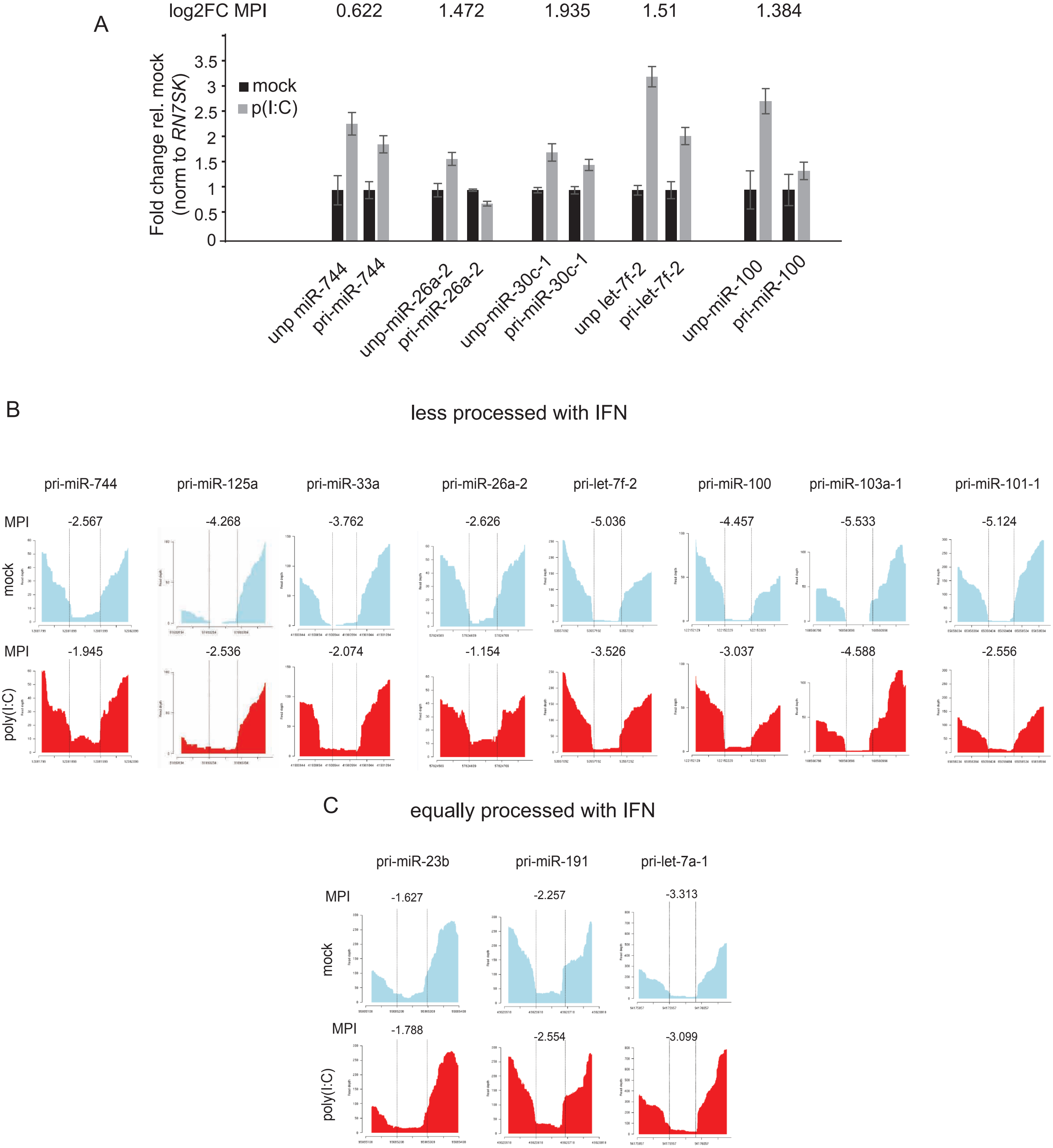

Figure S2. Validation of chromatin-associated pri-miRNA accumulation. Related to Figure 2. (**a**) Validation of chromatin associated RNA-high throughput sequencing by qRT-PCR. qPCR primers against unprocessed hairpins (unp) and transcript (pri) were designed for a total of 5 primary miRNAs (as in **Figure 2A**). Data shown is the average (n=4, biological replicates) +/- sem of chromatin-associated RNAs in mock, and in poly(I:C) treated conditions. Changes in processing efficiencies are at the top of the panel as log2FC MPI (**b**) RNA sequencing reads for chromatin associated RNA mock (light blue) or p(I:C) transfected samples (red) for pri-miRNAs negatively affected by the IFN response. Pre-miRNA hairpins are in the center, and extended +/-100 nt on each side. MPI values for each sample are shown at the top of each panel. (**c**) RNA sequencing reads for pri-miRNAs not affected by the IFN response, as in (**b**).

Figure S3

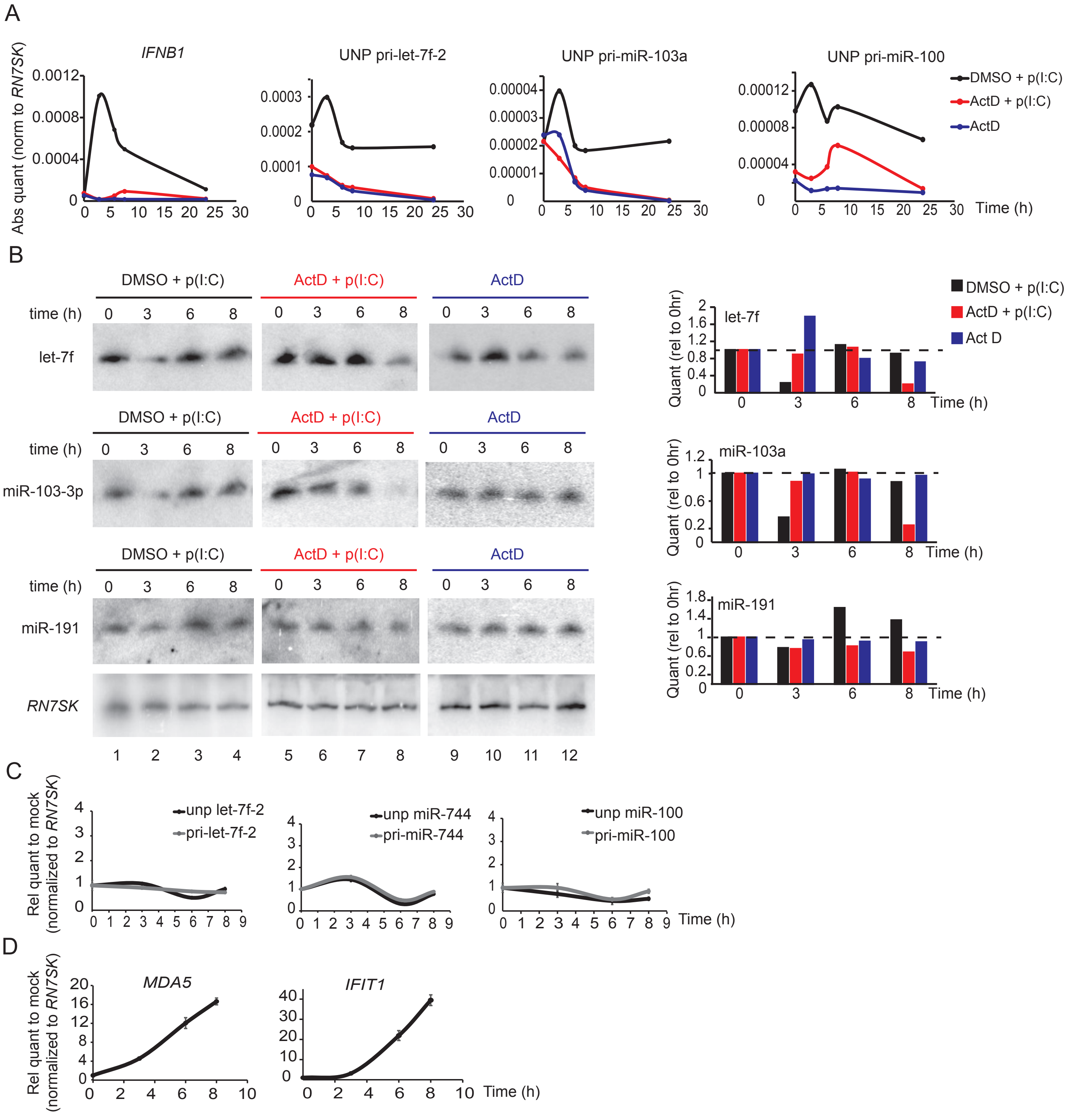

Figure S3. MiRNA stability during the IFN response. Related to Figure 2 and 3. (**a**) Time-course analysis for quantification of *IFNB1* mRNA and unprocessed pri-miRNAs (UNP) after incubation of HeLa cells with: DMSO + p(I:C) (in black), Actinomycin D (ActD) + p(I:C) (in red) and ActD (in blue). Absolute values normalized to *RN7SK* (an RNA-pol III transcript) are represented. (**b**) Northern analyses of timecourse as in (**a**) for let-7f (top), miR-103-3p (middle) and miR-191 (bottom), *RN7SK* serves as a loading control, with DMSO + p(I:C) (lanes 1-4), ActD + p(I:C) (lanes 5-8) and ActD alone (lanes 9-12). Northern quantifications are shown on the right, and expressed relative to 0hr time point, set as 1. (**c**) Time course analysis for quantification of unprocessed miRNA precursors (unp), and host transcripts (pri) after incubation of HeLa cells with media containing type-I Interferon, generated by poly(I:C) transfection in A549 cells. (**d**) The media used in (**c**) induces the expression of ISGs in HeLa cells: *MDA5*, and *IFIT1*. Data shown in (**c**) and (**d**) is the average value (n=3) +/- s.e.m.

Figure S4

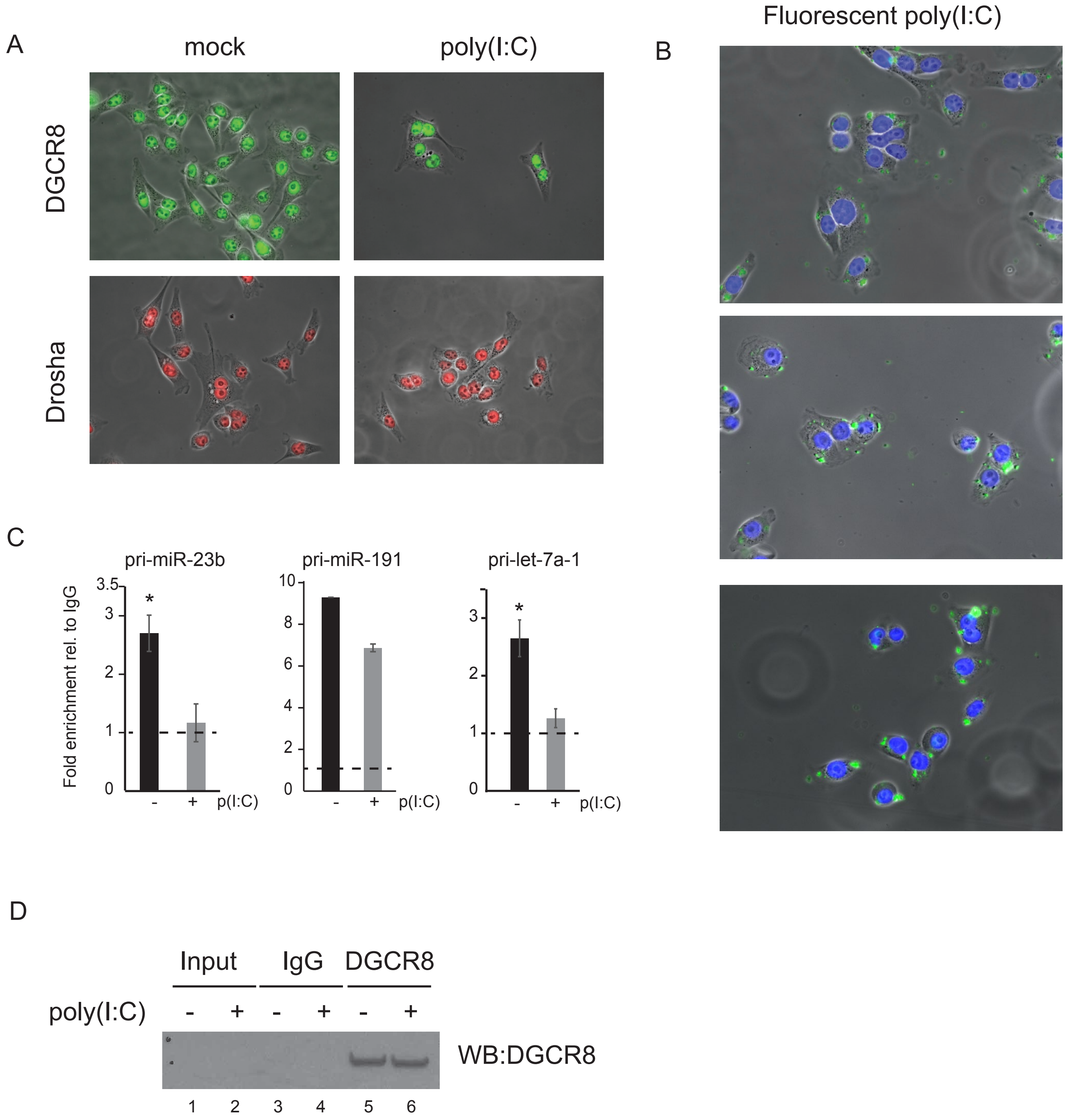

Figure S4. Altered DGCR8 binding to pri-miRNAs during IFN response. Related to Figure 4. (**a**) DGCR8 and Drosha remain nuclear during poly(I:C) transfection. Same as **Figure 4A** overlaid with bright-field microscopy (**b**) Fluorescently labeled poly(I:C) forms granules in the cytoplasm of HeLa cells (green), DAPI staining for nucleus (blue). (**c**) Quantification of pri-miRNA co-immunoprecipitated with endogenous DGCR8 in normal cells (black) and poly(I:C) transfected cells (grey). Data shown is the average of at least two experiments (n=2) +/- s.e.m, (*) p-value ≤0.05 when +/- poly(I:C) samples are compared, and relative to IgG control immunoprecipitation, set to 1 (dashed line). (**d**) Representative western blot analysis of immunoprecipitated endogenous DGCR8 in the absence (lane 5) and presence (lane 6) of poly(I:C).

Figure S5

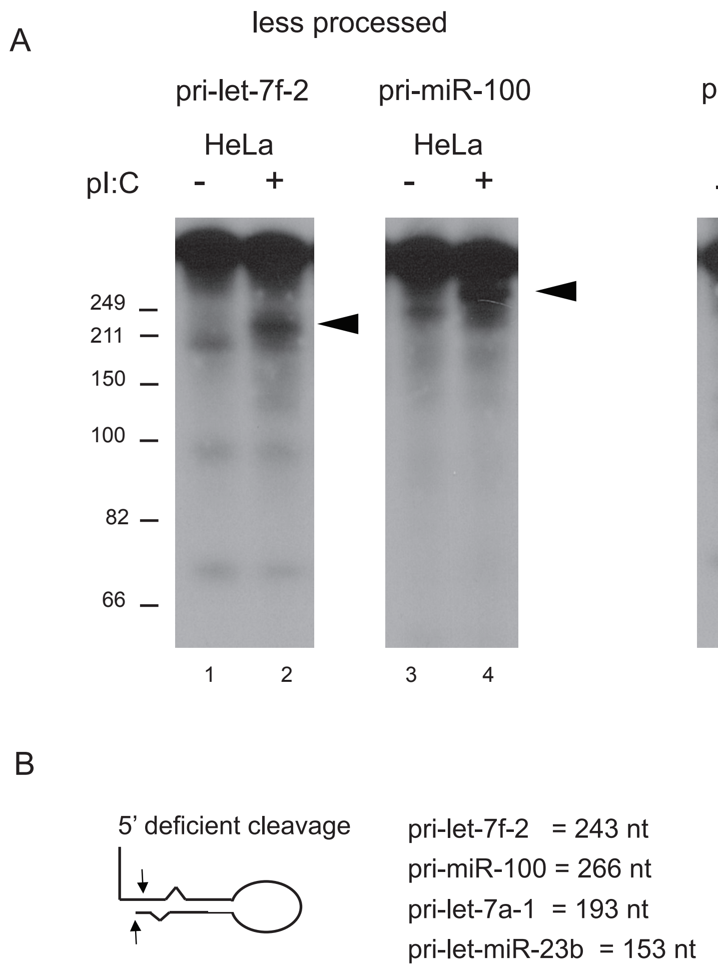

Figure S5. Accumulation of unprocessed pri-miRNAs upon IFN activation. Related to Figure 4. (**a**) *In vitro* processing assays of radiolabeled pri-let-7f-2 (lanes 1-2), pri-miR-100 (lanes 3-4), pri-let-7a-1 (lanes 5-6) and pri-miR-23b (lanes 7-8), with extracts from HeLa cells mock transfected (lanes 1, 3, 5 and 7), and poly(I:C) transfected (lanes 2, 4, 6 and 8). Black arrows indicate accumulation of processing intermediates (**b**) Schematic representation of pri-miRNAs failing to cleave the 5’end arm with their corresponding sizes in the substrates used in (**a**).

TableS1. Log2FC changes in pri-miRNA processing efficiencies upon poly(I:C) transfection in HeLa cells. Related to Figure 1.

Locus	MPI_mock	MPI dsRNA	Log2FC=MPI dsRNA -MPI mock
hsa-mir-125b-2	-4.928	-1.945	**2.983**
hsa-mir-101-1	-5.124	-2.556	**2.568**
hsa-mir-551b	-3.945	-1.43	**2.515**
hsa-mir-30c-1	-3.027	-1.092	**1.935**
hsa-mir-125a	-4.268	-2.356	**1.912**
hsa-mir-152	-2.488	-0.707	**1.781**
hsa-mir-33a	-3.762	-2.074	**1.688**
hsa-mir-107	-2.453	-0.813	**1.64**
hsa-let-7f-2	-5.036	-3.526	**1.51**
hsa-mir-567	-2.789	-1.301	**1.488**
hsa-mir-26a-2	-2.626	-1.154	**1.472**
hsa-mir-28	-1.624	-0.211	**1.413**
hsa-mir-100	-4.457	-3.073	**1.384**
hsa-mir-101-2	-4.438	-3.268	**1.17**
hsa-mir-30a	-5.012	-3.887	**1.125**
hsa-let-7c	-1.053	0.069	**1.122**
hsa-mir-556	-1.256	-0.145	**1.111**
hsa-mir-183	-2.112	-1.005	**1.107**
hsa-mir-31	-5.074	-4.024	**1.05**
hsa-mir-103a-1	-5.533	-4.588	**0.945**
hsa-mir-30e	-1.919	-0.997	**0.922**
hsa-mir-126	-1.451	-0.57	**0.881**
hsa-mir-324	-2.396	-1.56	**0.836**
hsa-mir-30d	-4.332	-3.552	**0.78**
hsa-mir-326	-1.479	-0.724	**0.755**
hsa-let-7a-2	-1.183	-0.446	**0.737**
hsa-let-7a-3	-3.242	-2.527	**0.715**
hsa-mir-182	-1.086	-0.423	**0.663**
hsa-mir-342	-3.043	-2.392	**0.651**
hsa-mir-425	-1.54	-0.894	**0.646**
hsa-mir-193b	-1.1	-0.469	**0.631**
hsa-mir-744	-2.567	-1.945	**0.622**
hsa-let-7g	-1.138	-0.521	**0.617**
hsa-mir-32	-1.741	-1.149	**0.592**
hsa-mir-423	-3.113	-2.523	**0.59**
hsa-mir-29b-2	-2.533	-1.949	**0.584**
hsa-mir-296	-0.973	-0.442	**0.531**
hsa-mir-548d-1	-1.547	-1.046	**0.501**
hsa-mir-221	-1.394	-0.896	**0.498**
hsa-mir-9-1	-3.537	-3.04	**0.497**
hsa-mir-24-1	-1.065	-0.636	**0.429**

**less processed**
**equally processed**
**more processed**

hsa-mir-29c	-0.443	-0.031		**0.412**
hsa-mir-641	-0.528	-0.142		**0.386**
hsa-mir-222	-3.687	-3.316		**0.371**
hsa-mir-30c-2	-0.987	-0.631		**0.356**
hsa-let-7f-1	-0.303	0.043		**0.346**
hsa-mir-301a	-2.312	-1.971		**0.341**
hsa-mir-582	-0.489	-0.16		**0.329**
hsa-mir-132	-0.092	0.225		**0.317**
hsa-mir-15b	-0.552	-0.245		**0.307**
hsa-mir-33b	-3.133	-2.838		**0.295**
hsa-mir-10a	-1.405	-1.112		**0.293**
hsa-mir-17	-2.073	-1.789		**0.284**
hsa-mir-22	-1.397	-1.118		**0.279**
hsa-mir-1307	-1.045	-0.796		**0.249**
hsa-mir-196b	-0.236	0.01		**0.246**
hsa-mir-26b	-3.384	-3.151		**0.233**
hsa-mir-331	-3.43	-3.206		**0.224**
hsa-let-7a-1	-3.313	-3.099		**0.214**
hsa-mir-30b	-5.8	-5.589		**0.211**
hsa-mir-671	-0.179	0.003		**0.182**
hsa-mir-98	-1.796	-1.66		**0.136**
hsa-mir-940	-0.208	-0.082		**0.126**
hsa-let-7d	-0.464	-0.339		**0.125**
hsa-mir-148b	-0.88	-0.763		**0.117**
hsa-let-7b	-1.267	-1.17		**0.097**
hsa-mir-29a	-0.952	-0.898		**0.054**
hsa-mir-1255a	-0.879	-0.827		**0.052**
hsa-mir-505	-0.288	-0.236		**0.052**
hsa-mir-27b	-0.891	-0.885		**0.006**
hsa-mir-579	-0.801	-0.803		**-0.002**
hsa-mir-151b	-0.13	-0.152		**-0.022**
hsa-mir-1304	-0.033	-0.059		**-0.026**
hsa-mir-628	-0.615	-0.642		**-0.027**
hsa-mir-548k	-0.168	-0.21		**-0.042**
hsa-mir-92b	-3.623	-3.678		**-0.055**
hsa-mir-193a	-0.943	-1		**-0.057**
hsa-mir-7-1	-0.573	-0.63		**-0.057**
hsa-mir-561	-0.095	-0.165		**-0.07**
hsa-mir-21	-1.827	-1.917		**-0.09**
hsa-mir-455	-0.238	-0.333		**-0.095**
hsa-mir-106b	-0.532	-0.628		**-0.096**
hsa-mir-576	-0.032	-0.14		**-0.108**
hsa-mir-298	-0.132	-0.24		**-0.108**
hsa-mir-130a	-3.502	-3.662		**-0.16**
hsa-mir-23b	-1.627	-1.788		**-0.161**
hsa-mir-374a	-0.982	-1.216		**-0.234**

hsa-mir-424	-0.1	-0.342	**-0.242**
hsa-mir-196a-2	-0.334	-0.588	**-0.254**
hsa-mir-34a	-1.37	-1.64	**-0.27**
hsa-mir-191	-2.257	-2.554	**-0.297**
hsa-let-7i	-0.264	-0.562	**-0.298**
hsa-mir-29b-1	-3.22	-3.552	**-0.332**
hsa-mir-503	-2.086	-2.486	**-0.4**
hsa-mir-20a	-0.542	-0.982	**-0.44**
hsa-mir-9-3	-3.735	-4.261	**-0.526**
hsa-mir-93	-0.744	-1.306	**-0.562**
hsa-mir-19b-1	-0.399	-1.012	**-0.613**
hsa-mir-103a-2	-2.445	-3.065	**-0.62**
hsa-mir-629	-1.889	-2.572	**-0.683**
hsa-mir-125b-1	-2.187	-4.109	**-1.922**
hsa-mir-96	-2.682	-4.614	**-1.932**
hsa-mir-16-2	-1.047	-4.152	**-3.105**

Table S3. **Complete list of oligonucleotide sequences used in this study. Related to Experimental Procedures.**

AGGCTGAAGATGGACACTGG	FORWARD unprocessed levels hsa-let-7f-2
CCACCGTGGGAAAGACAGTA	REVERSE unprocessed levels hsa-let-7f-2
TGGCAACCCCTACATTAGTCT	FORWARD transcriptional levels hsa-let-7f-2
GCCCAGAGAGCTACCTTCAT	REVERSE transcriptional levels hsa-let-7f-2
CTGGCTGCTTGGGTTCCT	FORWARD unprocessed levels hsa-miR-23b
ACGGTTTCTGGAGGAGCAG	REVERSE unprocessed levels has-miR-23b
CAGCTAGAATCTGCCTGGAGA	FORWARD transcriptional levels hsa-miR-23b
ATGCGACACACACAATTGCT	REVERSE transcriptional levels hsa-miR-23b
TGTCTCCAGAGCATTCCAGC	FORWARD unprocessed levels hsa-miR-191
GAGACCCAAGCAGCTCAGTA	REVERSE unprocessed levels hsa-miR-191
CCCCACCTCCCCTTCTATTG	FORWARD transcriptional levels hsa-miR-191
ACATCAGAACCCGCCCATC	REVERSE transcriptional levels hsa-miR-191
GGAAGAAGACGCAGCACAC	FORWARD unprocessed levels hsa-miR-744
AGTAAGGTTGAGGTTAGTGGCA	REVERSE unprocessed levels hsa-miR-744
TGTTGTGTTCCTACTTTTGTGGT	FORWARD transcriptional levels hsa-miR-744
GCAATCAACATGCACACTTCA	REVERSE transcriptional levels hsa-miR-744
TTCATGGGGAGCCTTCAGAG	FORWARD for unprocessed levels pri-miR-101-1
CAGCACTGTGATAACTGAGCC	REVERSE for unprocessed levels pri-miR-101-1
TTTCTTCTGCCTCCTCACGT	FORWARD for transcriptional levels pri-miR-101-1
GGTCAACGGCATCCTTTCTG	REVERSE for transcriptional levels pri-miR-101-1
ATGTCACAGCCCCAAAAGAG	FORWARD pri-miR-100 unprocessed levels
CCACAAGTTCGGATCTACGG	REVERSE pri-miR-100 unprocessed levels
AGGAATGGACTTGCATTTGG	FORWARD pri-miR-100 transcription levels
AAATGGCAAACCAGCAGAAT	REVERSE pri-miR-100 transcription levels
TGCTCACCACTATACCACCA	FORWARD for unprocessed levels human pri-miR-103-1
TAGCCCTGTACAATGCTGCT	REVERSE for unprocessed levels human pri-miR-103-1
ACACAACCTAAATCCCTTGAGG	FORWARD for transcriptional levels pri-miR-103-1
AGCTGCCTTCCAAATGCAAA	REVERSE for transcriptional levels pri-miR-103-1
ACACCCACCACTGGGAGATA	FORWARD for unprocessed pri-let-7a-1 levels
GCCTGGATGCAGACTTTTCT	REVERSE for unprocessed pri-let-7a-1 levels
GCATTTGTTTATGGCCTGGA	FORWARD for pri-let-7a-1 transcriptional levels
CACCCCCATCCAGTGTACTT	REVERSE for pri-let-7a-1 transcriptional levels
CCAAGGACAGAAAGCTCCCA	FORWARD unprocessed levels hsa-miR-26a-2
TGCCTCCAGAAACAAGTAATCA	REVERSE unprocessed levels hsa-miR-26a-2
AGGCTAGAGTCATGGAAGCA	FORWARD transcriptional levels hsa-miR-26a-2
CCGATCTTGTGCTTGCTTCT	REVERSE transcriptional levels hsa-miR-26a-2
CTGATCAACCCTGGACCCTG	FORWARD unprocessed levels hsa-miR-30c-1
TAAACAACCCTCTCCCAGCC	REVERSE unprocessed levels hsa-miR-30c-1
TACTTTGGGGAGTTGGAGGC	FORWARD transcriptional levels hsa-miR-30c-1
ACTTGGGCTGGCTGAGTAAA	REVERSE transcriptional levels hsa-miR-30c-1
TGAGGTAGTAGATTGTATAGTTCCTGTCTC	Northern probe against mir-let-7f-5p
AGCAGCATTGTACAGGGCTATGACCTGTCTC	Northern probe against miR-103a-3p

TACAGTACTGTGATAACTGAACCTGTCTC	Northern probe against miR-101-1-3p
CAACGGAATCCCAAAAGCAGCTGCCTGTCTC	Northern probe against miR-191
CCTGCTAGAACCTCCAAACAAGCCTGTCTC	Northern probe against *RN7SK*
CCTGAAGGCCAAGGAGTACA	FORWARD human *IFNB1* mRNA quantification
AGCAATTGTCCAGTCCCAGA	REVERSE human *IFNB1* mRNA quantification
CTCTCTCTAATCAGCCCTCTGG	FORWARD human *TNFA* mRNA quantification
GTTGACCTTGGTCTGGTAGGAG	REVERSE human *TNFA* mRNA quantification
TCCAGAATCGAAGGCCATCA	FORWARD human *CXCL10* mRNA levels
CTTGGAAGCACTGCATCGAT	REVERSE human *CXCL10* mRNA levels
GTCTGGGGCATGGAGAATAA	FORWARD human *MDA5* mRNA
TGCCCATGTTGCTGTTATG	REVERSE human *MDA5* mRNA
TCTCAGAGGAGCCTGGCTAAG	FORWARD human *IFIT1* mRNA
CCACACTGTATTTGGTGTCTAGG	REVERSE human *IFIT1* mRNA
ACTCTCCTTCCCTTTCTCCC	FORWARD to amplify -100 pri-let-7f-2 and clone in pGEMt
TGAACAAGACACATGACCTCA	REVERSE to amplify +100 pri-let-7f-2 and clone in pGEMt
GCTTTTGTGAGAGTGGGACG	FORWARD to amplify pri-miR-100 cloning in pGEMt
GCATATAAGCAAAGCCCCAGG	REVERSE to amplify pri-miR-100 cloning in pGEMt
CTTCCTGTGGTGCTCAACTG	FORWARD to amplify pri-let7a1 cloning in pGEMt
GGCCATAAACAAATGCTGCAC	REVERSE to amplify pri-let7a1 cloning in pGEMt
taatacgactcactatagggGTGAAGGCGGCAGTGTCGCCGG	FORWARD T7 promoter fused to -100nt pri-miR-23b for in vitro transcription
TGGTGGCTTCTTTTGTTTCCA	REVERSE for pri- hsa-miR-23b in vitro transcription template
